# Real-time depth completion based on LiDAR-stereo for autonomous driving

**DOI:** 10.3389/fnbot.2023.1124676

**Published:** 2023-04-18

**Authors:** Ming Wei, Ming Zhu, Yaoyuan Zhang, Jiarong Wang, Jiaqi Sun

**Affiliations:** ^1^Changchun Institute of Optics, Fine Mechanics and Physics, Chinese Academy of Sciences, Changchun, China; ^2^University of Chinese Academy of Sciences, Beijing, China

**Keywords:** sensor fusion, depth completion, point cloud, autonomous driving, LiDAR-stereo

## Abstract

The integration of multiple sensors is a crucial and emerging trend in the development of autonomous driving technology. The depth image obtained by stereo matching of the binocular camera is easily influenced by environment and distance. The point cloud of LiDAR has strong penetrability. However, it is much sparser than binocular images. LiDAR-stereo fusion can neutralize the advantages of the two sensors and maximize the acquisition of reliable three-dimensional information to improve the safety of automatic driving. Cross-sensor fusion is a key issue in the development of autonomous driving technology. This study proposed a real-time LiDAR-stereo depth completion network without 3D convolution to fuse point clouds and binocular images using injection guidance. At the same time, a kernel-connected spatial propagation network was utilized to refine the depth. The output of dense 3D information is more accurate for autonomous driving. Experimental results on the KITTI dataset showed that our method used real-time techniques effectively. Further, we demonstrated our solution's ability to address sensor defects and challenging environmental conditions using the p-KITTI dataset.

## 1. Introduction

The key to autonomous driving technology is ensuring safety during driving. Awareness of the surrounding environment is the basis of various intelligent strategies. Intelligent cars require the comprehensive analysis of data from various sensors to accurately perceive their surrounding environment while in motion. Therefore, cross-sensor information fusion technology is a vital method of improving the ability of 3D information acquisition (You et al., 2020; Kim et al., 2022). In addition, enhancing the perception of sensors could improve the safety and stability of automatic driving. It represents the mainstream direction of development (Cui, 2022; Eom and Lee, 2022).

LiDAR-stereo has been gradually developed in recent years (Cholakkal et al., 2020). LiDAR has strong penetrability and can directly generate effective point clouds. It is less affected by environmental disturbances. It consumes less space and is more robust and stable. However, the LiDAR point cloud of a low-power laser beam is cost-effective but sparse. Additional information from other sensors can compensate for the loss of 3D data (Nickels et al., 2003; Badino et al., 2011). Therefore, cross-sensor fusion is essential for depth completion tasks. Cameras are selected as auxiliary sensors because of their low costs and simple structure. At present, deep completion networks are based on the fusion of a monocular camera and LiDAR. A monocular camera provides a single sheet of rich color and edge information for sparse point clouds. According to this supplementary information, sparsity and incomplete point clouds can be improved. However, the information provided by monocular images is limited, as they do not provide true depth information. The completion results are usually not reliable enough (Xu and Zhang, 2020; Hu et al., 2021). As for binocular cameras, the disparity image is obtained by stereo matching (Kendall et al., 2017; Huang et al., 2020). Binocular cameras have all the advantages of monocular cameras and contain real 3D information. Therefore, LiDAR-stereo is the development trend for future automatic drive sensors.

The LiDAR-stereo system provides vital 3D information for automatic driving. When point clouds and binocular images are used in the depth completion task, the integrality of 3D information can be improved through the integration of rich data. This process transforms the sparse and incomplete 3D information obtained from LiDAR into a denser and more effective representation (Park J. et al., 2020; Zhao et al., 2021; Wei et al., 2022). However, the current effective method is to structure two or three branches for the point cloud and binocular image. Features are fused by constructing the feature cost volume. In addition, 3D convolution is utilized to extract the overall features (Tran et al., 2014). Although 3D convolution is effective, it takes up huge amounts of time and space. It loses real-time performance. The speed of 3D information acquisition by intelligent vehicles while driving needs to be guaranteed. To find an alternative to 3D convolution, HIT-Net was proposed by Tankovich et al. (2021) as an efficient hierarchical iterative tiling concept to infer disparity assumptions. It successfully improves the speed of stereo matching. Based on the idea of iterative tiling, we designed a new real-time depth completion network based on LiDAR-stereo that replaces 3D convolution with propagated 2D convolution to obtain dense depth quickly. Unlike the original parallel structure of other fusion networks, we combined point clouds and stereo-depth images using the multi-injection method. Point clouds were injected multiple times to guide the refinement of depth information stage by stage. It compensated for the errors caused by confusion. In addition, we designed a convolutional space propagation network based on kernel connections to further optimize the depth. The traditional multi-core spatial propagation network adopts a parallel structure. There was no connection between the different cores. Large convolution kernels lost some detail at each propagation. Our structure was able to avoid this phenomenon and expand the network width to improve results.

## 2. Related works

Depth sensor and binocular camera data fusion technologies were initially studied in the field of robotics (Nickels et al., 2003). They generally adopt the fusion methods of stereo and TOF cameras. However, because of the uncertainty of the outdoor environment, these methods cannot be transferred and generalized to the field of autonomous driving. Researchers have proposed directly integrating LiDAR data into stereo algorithms to reduce errors and to increase the density of texture-free regions (Badino et al., 2011). However, the data obtained through this approach are limited and lack generalizability. In traditional methods, probabilistic fusion combines prior information from each sensor and introduces a probabilistic model that integrates LiDAR and binocular data. They fuse sparse point clouds with stereo images to provide accurate, dense-depth images and real-time uncertainty estimates (Maddern and Newman, 2016). However, the performance dropped significantly in areas lacking a point cloud. With the rise and development of deep learning, neural networks were utilized to complete 3D information based on multi-source information fusion.

### 2.1. LiDAR-mono fusion

The basic framework of a color image and point cloud fusion is widely used in many depth completion tasks. The color image serves as a helpful guide to refine the depth image as supplementary information. One approach, known as Convolutional Spatial Propagation Network (CSPN), employs cyclic convolution operations to propagate and refine the depth image by learning the affinity between the adjacent pixels through a deep convolutional neural network (Cheng et al., 2018, 2019a). In addition, CSPN integrates sparse-depth samples into the propagation process and employs 3D convolution to generate a dense depth map. Furthermore, CSPN++ further improves its effectiveness and efficiency by learning the adaptive convolution kernel size and propagation iteration times (Chen et al., 2018). Spade was proposed as a sparse-depth data processing method with optional dense RGBS that can effectively learn sparse features without the additional validity masks (Jaritz et al., 2018). Ma et al. (2019) proposed Sparse2Dense++ as a method to develop a self-supervised training framework and deep regression model to learn the direct mapping from sparse depth and color images to dense depth. Chen et al. (2018) designed a depth estimation model that is robust to common measurement errors for both indoor and outdoor scenes. They chose a pre-fusion strategy. NConv-CNN processes the image and sparse-depth mapping in parallel and utilizes normalized convolution to handle the highly sparse depth and confidence (Eldesokey et al., 2020). Fusion-Net improves accuracy to account for confidence masks for the uncertainty in each mode of depth prediction (Van Gansbeke et al., 2019). DeepLiDAR estimates the surface normal as an intermediate representation to produce dense depth. It predicts a confidence mask to handle mixed LiDAR signals that occlude near the foreground boundary (Qiu, 2019). Estimating color images and surface normals is combined with the learned attention map to improve depth accuracy at long distances. DDP was proposed as a prior conditional network to associate probabilities with each given depth value through probabilistic priors of depth (Yang et al., 2019). It is combined with a likelihood term using sparse measurements. However, because monocular cameras cannot directly obtain 3D information, these networks mostly utilize the color, texture, and area information of images, which cannot truly compensate for the missing depth.

### 2.2. LiDAR-stereo fusion

Binocular images have more information than monocular images. The disparity can be obtained directly through the left and right image pairs. The most difficult part is fusing 3D information from multi-source sensors.

Cost volume was widely utilized in processing non-single input to fuse information from two images or multiple sources (Zhang et al., 2019). Therefore, most LiDAR-stereo fusion methods build a variety of cost volumes and then refine the depth in different ways. One notable example is the CCVNorm approach, which involves fusing point cloud information and applying conditional cost volume normalization to enhance the fusion effect (Wang et al., 2019). Another method, SDC-Net, employs a unique hierarchical and geometrically inspired framework for deep completion learning (Choi et al., 2021). The virtual right image was inferred from the left image and point cloud, leading to depth completion by simulating stereo matching. However, it adds to the complexity of the network. Mai et al. (2021) proposed SLS-Fusion, a sparse LiDAR and stereo fusion network, and applied it to 3D object detection. They proposed multiple jump fusions to gradually construct the cost volume. Sparse and accurate point clouds guide the correspondence of stereo images in a unified 3D volume space. Unlike the existing fusion strategies, VPN directly embeds the point cloud into the cost volume, which can disseminate effective information to the nearby voxels in the cost volume and reduce uncertainty (Choe et al., 2021). FastFusion was proposed as a binary neural network integrating stereo-matching information as input (Meng et al., 2021). Meng et al. fused stereo-matching data and sparse point cloud data-based LiDAR aggregation. It is a two-stage fusion network. The networks mentioned above achieve better results. However, they built the cost volume that needs 3D convolution to extract multi-source features. It consumes a large amount of time and space. Real-time performance cannot be guaranteed in real-time autonomous driving.

Therefore, using new fusion and optimization methods to replace cost volume is key to solving the accuracy and speed problems of deep completion networks based on LiDAR-stereo fusion. In addition to constructing cost-effective volumes, researchers have recently attempted other methods to quickly fuse LiDAR and stereo with less time consumption. CenterFusion was proposed as an intermediate fusion method that uses a frustum-based approach to correlate LiDAR detection with the central point of the corresponding target. A point cloud feature map is used to supplement image features and improve the accuracy of 3D target detection (Nabati and Qi, 2020). MVAF-Net was proposed as an attentive pointwise weighting module and helps to learn structure information by an adaptive fusion method of multi-view features (Wang et al., 2020). HDE-Net directly combines point cloud results from the LiDAR and the semi-global matching results from the binoculars. It encodes the complementary features of sparse 3D LiDAR and dense stereo depth in an enhanced manner (Park K. et al., 2020). However, it is not an end-to-end network. LiDAR-stereo-Net was proposed as an unsupervised and pre-fusion network (Cheng et al., 2018, 2019b). The input of the network is the image pairs and sparse-depth images. It solves the problem of misalignment between LiDAR noise points and binocular sensors by introducing a new ”feedback loop“ to connect a network of inputs and outputs. LiDAR-stereo was proposed as a self-supervised training method to obtain effective depth information. It adopted the feature fusion strategy and only merged three kinds of feature information in the fusion stage of the network (Zhang et al., 2020). SLF-Net generated a coarse disparity image by fusing a point cloud projection with a color image (Zhang et al., 2022). Stereo fusion and edge sense refinement make the depth discontinuities consistent with the edges in the image. It relies excessively on the accuracy of color images. Once the target was obscured, the edge-sensing optimization lost its effect. Although the speed of these networks was higher, the accuracy was not good enough with the single fusion mode. Our network (RLS-Net) was proposed as a parallel fusion to replace lost volume and improve effectiveness from global and local perspectives. The advantages were as follows:

(1) We designed a new LiDAR-stereo depth completion network for autonomous driving. The multi-injection method guides multi-source 3D information fusion and updating to improve the effectiveness of global depth.
(2) We proposed a kernel-connected convolutional spatial-propagation network. Parallel independent convolution kernels were concatenated to expand the network width and refine the edges of local depth.
(3) The experiments on the KITTI dataset showed that the dense and complete depth image obtained by our real-time network works well.

## 3. Methodology

**Figure 1:**
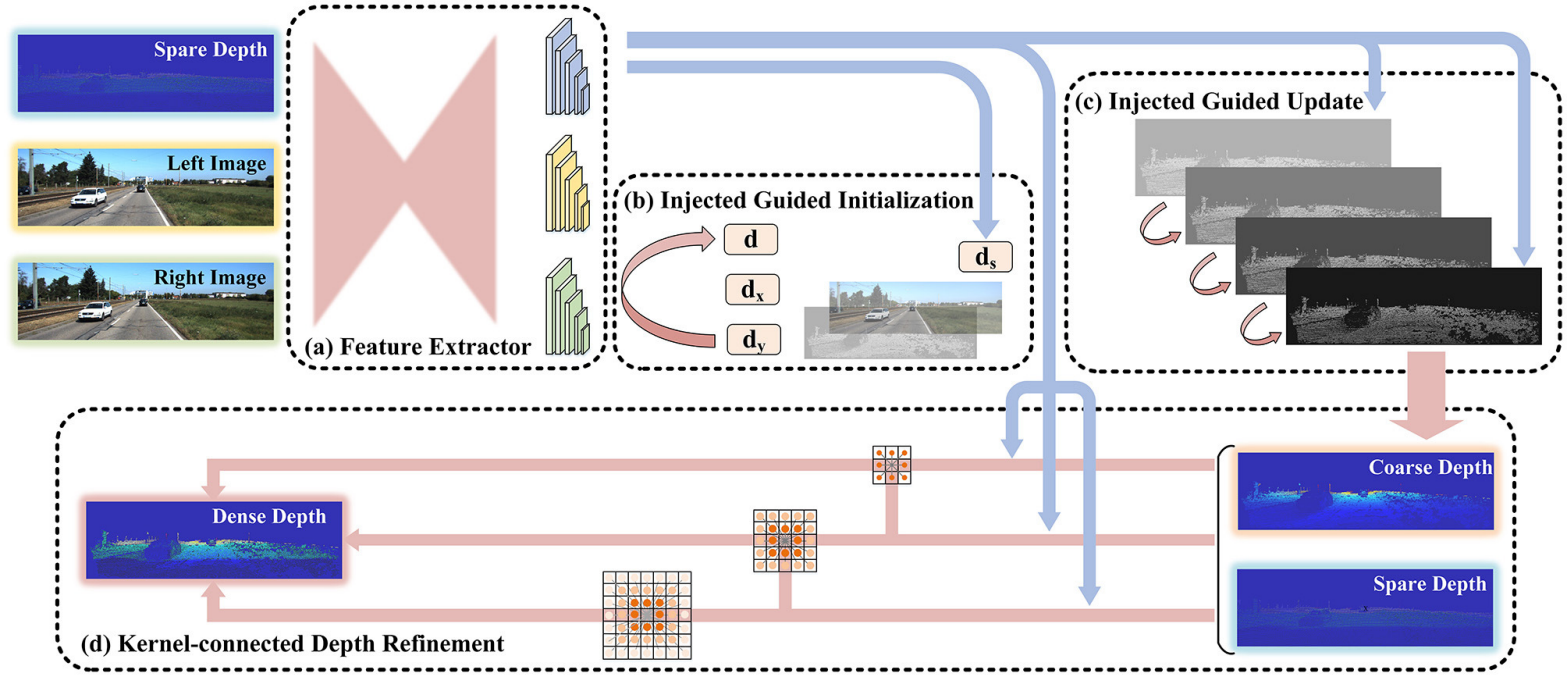
The structure of our network. The inputs of the network are the sparse depth image from LiDAR and the image pair from the binocular camera. The output is the dense depth image. Initially, the multi-source images get the corresponding features through the feature extractor with shared weight. Then, the coarse depth is obtained by initializing and iteratively updating the depth information under the guidance of the features of the point cloud. Finally, the depth is optimized in the refinement module to obtain the dense depth image.

The structure of our network is shown in Figure 1. The network consists of four modules: the feature extractor module, the injected guided initialization module, the injected guided update module, and the kernel-connected depth refinement module. The feature extractor adopted a U-shaped structure. Weights were shared based on similarities in information from multiple sources. The multiple injection guidance module is described in Sections 3.1.1 and 3.1.2. The point cloud information was initialized, and the guided depth was updated according to the injection strategy. In addition, we proposed a spatial propagation network of kernel connections to optimize depth, which is described in Section 3.2.

The data flow process in the network is as follows: first, the sparse-depth image was obtained by mapping the 3D point cloud to the 2D plane that aligns with the binocular image. Second, we transformed three images from different sources into three feature images through the feature extraction module. The features were then fused into the following injection guide module and estimated to be a coarse-depth image. Finally, the depth image was improved using the refinement module.

### 3.1. Multiple injected guidance

HIT-Net was proposed for multi-resolution initialization, differentiable 2D geometry propagation, and bending mechanisms to accelerate and replace 3D convolution (Tankovich et al., 2021). It effectively avoids the consumption of the cost. We applied similar ideas to LiDAR-stereo fusion networks that completed depth information. As shown in (b) and (c) of Figure 1, sparse-depth images guided the fusion of information from multiple sources with injections. It consisted of two steps: initialization and updating.

#### 3.1.1. Initialization

The feature extractor converted sparse point cloud images *S* ∈ ℝ^*B*, 1, *H, W*^ and binocular images *L, R* ∈ ℝ^*B*, 3, *H, W*^ into feature images $F_{j}\in {\mathbb{R}}^{B,C_{j},H_{j},W_{j}}$, where ℝ is the dimension domain of the feature, *B* is the batch size, *C* is the number of channels, *H* and *W* are the width and height. *j* represents the structure of multiple resolutions. The three types of information were still image-based data structures with multiple channels. However, it saved the feature representation rather than the pixel information. The feature images were preprocessed into the required structure to meet the initialization needs. They can be expressed as


(1) $$\begin{array}{l} \sigma_{i}^{s}\in {\mathbb{R}}^{B,C_{s},\frac{H}{2^{i+1}},\frac{W}{2^{i+1}}},\sigma_{i}^{l}\in {\mathbb{R}}^{B,C_{l},\frac{H}{2^{i+1}},\frac{W}{2^{i+1}}},\sigma_{i}^{r}\in {\mathbb{R}}^{B,C_{r},\frac{H}{2^{i+1}},W} \end{array}$$

We represent the feature vectors as a confluent tile hypothesis with autonomous learning ability. Unlike the old construction, we considered the sparsity of the point clouds, which can be regarded as a concrete representation of the relative absence and sparsity of the point cloud. We innovatively obtained binocular images and point clouds while also measuring the difference in gradients between them to formulate a testable hypothesis.

**Figure 2:**
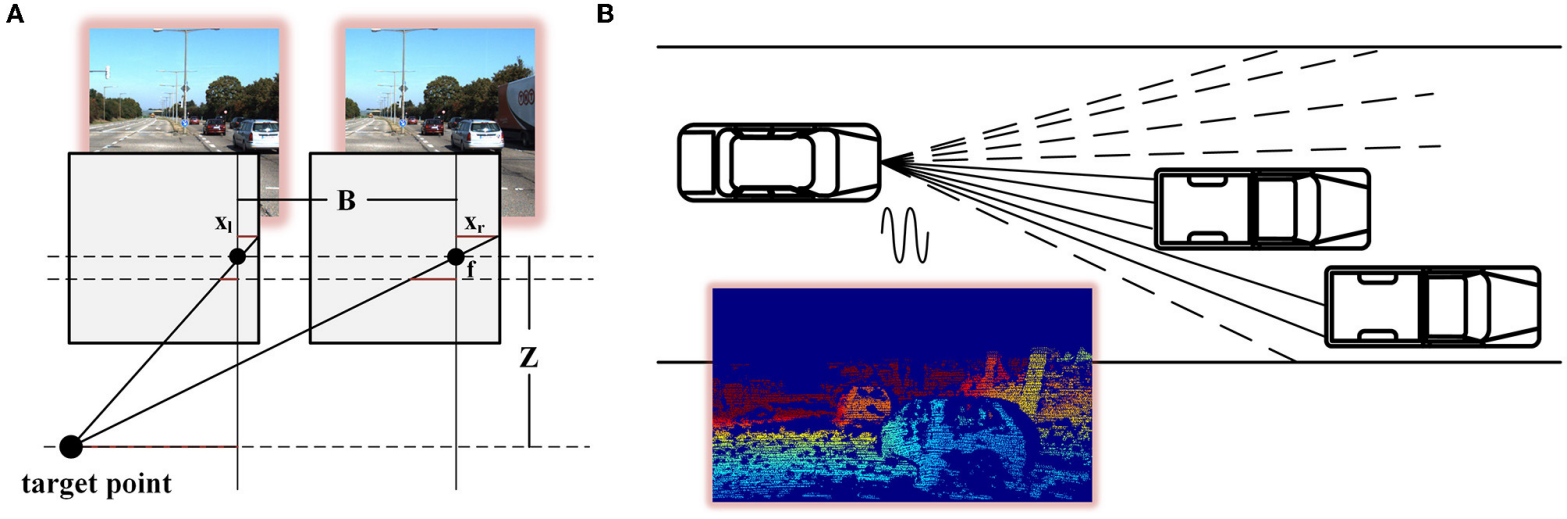
Depth measurement model of stereo matching and point cloud scanning. **(A)** is the model of a binocular camera, the lower dot is the target point, and the upper two dots are the two cameras **(B)**.

The initial depth of the binocular images and the initial sparsity of the point cloud can be obtained from the physical model. As shown in Figure 2A, the corresponding disparity can be obtained for stereo vision by finding the corresponding points in the scene of the l binocular images. The depth of the scene can be calculated from the baseline *b* and focal length *F*, which is $Z=\frac{bf}{d}$. Because there was a one-to-one correspondence between depth and disparity, depth could be represented by the disparity in deep learning networks. Feature images of different scales were obtained using the multi-resolution feature extractor for shared weights (a specific combination of complex convolution). According to the feature information, the initial binocular disparity defined by one norm concerning HIT-Net ${z_{i}}^{0}$ can be expressed as


(2) $$\begin{array}{l} z_{i}^{0}\in {\mathbb{R}}^{B,C_{d},\frac{H}{2^{i+1}},\frac{W}{2^{i+1}}}=\\ \arg\min{\left\|\overset{l}{\underset{i}{\sigma }}\in {\mathbb{R}}^{B,C_{l},\frac{H}{2^{i+1}},\frac{W}{2^{i+1}}}-pad\left(\sigma_{i}^{r}\right)\in {\mathbb{R}}^{B,C_{r},\frac{H}{2^{i+1}},W}\right\|}_{1} \end{array}$$

where *pad*() is the fill method.

However, as shown in Figure 2B, the vehicle-borne LiDAR components handled the vehicle-borne LiDAR components emitting lasers within a field angle. At the same time, the receiving components received the laser that was reflected within the receiving range. Information about the reflected object can be obtained from the correlation between the emitted laser and the reflected laser. Therefore, the point cloud formed by LiDAR was composed of points. The depth of information was reliable. However, the fewer beams of LiDAR, the lower the point cloud density. 3D information could be effectively identified. We processed the point cloud with the same feature extractor to obtain the sparse multi-resolution feature of the point cloud. The point cloud corresponded to the left image, and the sparsity gradient ${s_{i}}^{0}$ was defined as


(3) $$\begin{array}{l} {s_{i}}^{0}\in {\mathbb{R}}^{B,C_{s},\frac{H}{2^{i+1}},\frac{W}{2^{i+1}}}=\\ cat\left(\sigma_{i}^{l}\in {\mathbb{R}}^{B,C_{l},\frac{H}{2^{i+1}},\frac{W}{2^{i+1}}},\sigma_{i}^{s}\in {\mathbb{R}}^{B,C_{s},\frac{H}{2^{i+1}},\frac{W}{2^{i+1}}}\right) \end{array}$$

where *cat*() is the connection method.

In addition, the closer to the depth of the real multi-source sensor, the better the fusion effect. From the perspective of sensor fusion, is the correspondence between multi-source information and the fusion state of LiDAR and binocular. The difference between the x and y directions was set to 0 in the initialization, which is ${d_{ix}}^{0}={d_{iy}}^{0}=0$. They were updated and refined through subsequent cycles.

The initial tile hypothesis consisted of four vectors. We set the number of resolution layers as *N* and the scale as *i* ∈ 1, ⋯  , *N*. Therefore, combining these feature vectors above, the initial tile hypothesis of scale *i* is defined as


(4) $$\begin{array}{l} {T_{i}}^{0}\in {\mathbb{R}}^{B,C,\frac{H}{2^{i+1}},\frac{W}{2^{i+1}}}=\left\{{z_{i}}^{0},{s_{i}}^{0},{d_{ix}}^{0},{d_{iy}}^{0}\right\} \end{array}$$

where ${z_{i}}^{0}$ is the initial depth of the binocular, ${s_{i}}^{0}$ is the sparsity of the point cloud. ${d_{ix}}^{0}$ and ${d_{iy}}^{0}$ are the ladder differences between the two in the *x* and *y* directions.

#### 3.1.2. Updating

**Figure 3:**
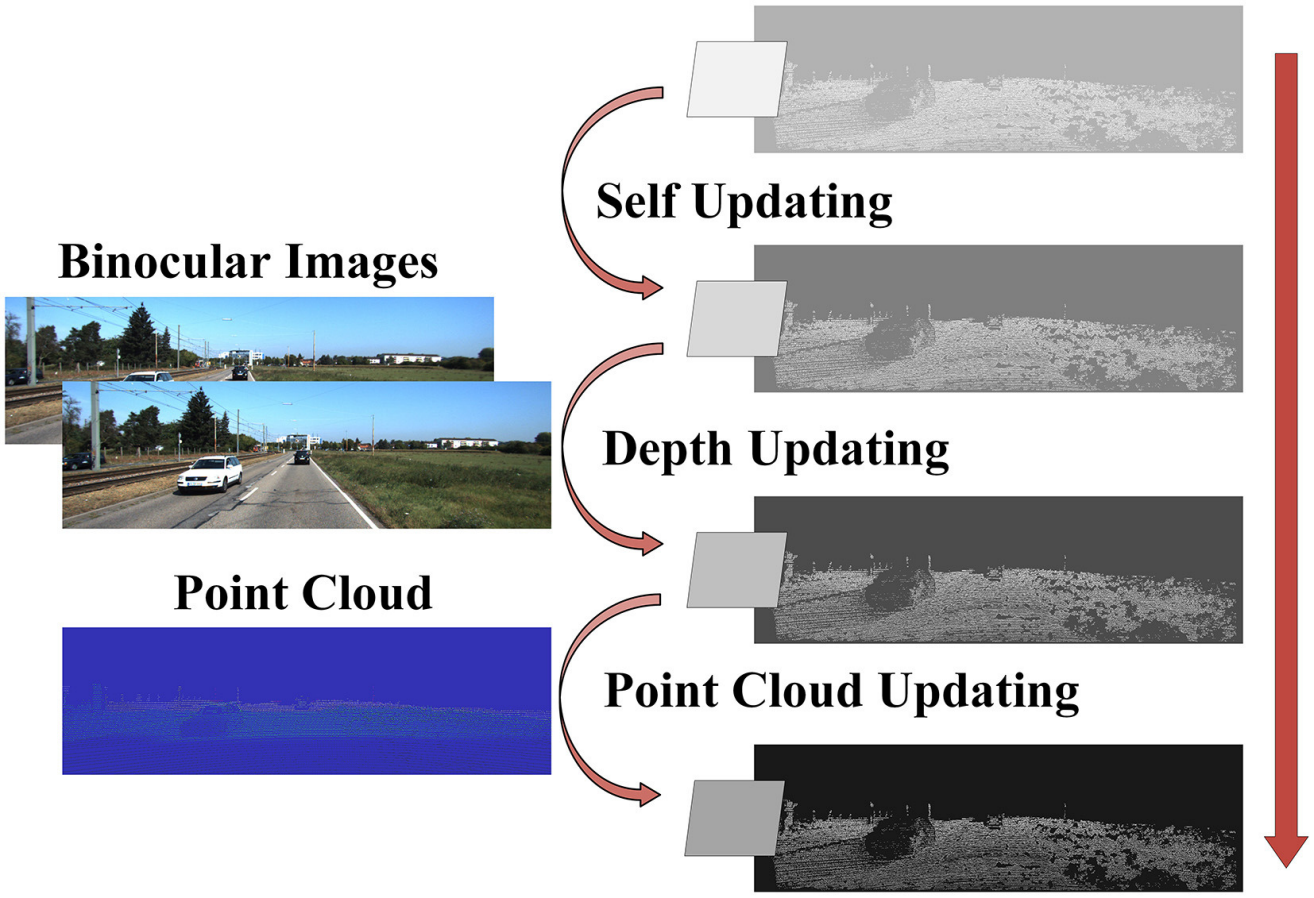
Schematic diagram of triple updating. Tile hypotheses are the majority of feature representation and update for depth images. The arrow from top to bottom in the direction of the update. The binocular image is injected in the depth updating. The point cloud is incorporated into the point cloud updating.is the model of LiDAR, the lines are waves.

Tile hypotheses are gradually improved through spatial communication and information fusion in the updating process. It consisted of three steps: self-updating ${{Ṫ}_{i}}^{m}$, depth updating ${\ddot{T}}_{i}^{m}$, and point cloud updating ${\overset{\mathrm{...}}{T}}_{i}^{m}$. It is shown in Figure 3.

Initially, because of the sparsity of the point cloud, there may be some error in the difference between the point cloud and the binoculars in the iteration. On the contrary, the overall information in the binocular images was denser and more reliable. Therefore, for the self-updating process, we focused on the depth disparity of the same tile hypotheses. Disparity and gradients in the *x* and *y* directions were updated. In the local 4 × 4 windows, the self-updating result ${{Ṫ}_{i}}^{m}$ was obtained by collecting gradients in *x* and *y* directions. The tile hypotheses-based self-updating can be expressed as


(5) $$\begin{array}{l} {{Ṫ}_{i}}^{m}\left\{d,d_{ix},d_{iy}\right\}\in {\mathbb{R}}^{B,C,\frac{H}{2^{i+1}},\frac{W}{2^{i+1}}}=\\ \sum {\underset{h,w}{win}}^{{\;}_{4\times 4}}\left\{d+d_{ix}\left(h-\frac{3}{2}\right)+d_{iy}\left(w-\frac{3}{2}\right)\right\}, \end{array}$$

where *m* is the update times, and *win*() is the local setting window.

Subsequently, for binocular images, we scaled the hypotheses tiles to match the feature pyramid's scale. The output was based on the left image. Therefore, the right image was biased concerning depth, and the left image was consistent. Each pixel in the output image was located at the corresponding pixel point in the input image according to the flow value. This process is known as “warping” in optical flow. We mapped the virtual optical flow value represented by the feature image on the right with the photometric consistency. The tile hypotheses based on depth updating ${\ddot{T}}_{i}^{m}$ can be expressed as


(6) $$\begin{array}{l} {{\ddot{T}}_{i}}^{m}\in {\mathbb{R}}^{B,C,\frac{H}{2^{i+1}},\frac{W}{2^{i+1}}}={\underset{h,w}{conv}}^{4\times 4}\left\{\sigma_{i}^{l},flow\left({{Ṫ}_{i}}^{m},\sigma_{i}^{r}\right)\right\}=\\ {\underset{h,w}{conv}}^{4\times 4}\left\{\sigma_{i}^{l},\left({{Ṫ}_{i}}^{m}\left\{h+u,w+u\right\}\right)\right\}, \end{array}$$

where *flow*() is the wrap method of optical flow, *conv*() is convolution, $\sigma_{i}^{l}$ the left feature image, $\sigma_{i}^{r}$ is the right feature image, and *u* is the offset of the mapping.

Finally, we added point cloud information using the injection method. The reliability of the information decreased gradually due to the upsampling, which caused the resolution to increase gradually. Therefore, we added point cloud features gradually in the process of upsampling to optimize the high-resolution information. The tile hypotheses based on point cloud updating can be expressed as


(7) $$\begin{array}{l} {{\overset{\mathrm{...}}{T}}_{i}}^{m}\in {\mathbb{R}}^{B,C,\frac{H}{2^{i+1}},\frac{W}{2^{i+1}}}=\;cat\;\left({\ddot{T}}_{i}^{m},\sigma_{i}^{s}\right) \end{array}$$

The coarse-depth image $D_{ori}\in {\mathbb{R}}^{B,1,H,W}$ can be obtained through three update steps, and the number of iterations can be set freely. However, the resolution changes cause the local information to be unstable. Therefore, we opted to do local optimization.

### 3.2. Kernel-connected depth refinement

CSPN was proposed to enhance local depth information. It utilizes an anisotropic diffusion process. It learns from a specific image directly using deep convolution to fuse neighborhood information and improve efficiency. The coarse-depth image was put into the module. Moreover, the data structure of the intermediate variable remained the same during the process. In addition, the number of channels varied according to the size of the convolution kernel. The iterative formula of convolutional space propagation can be expressed as


(8) $$\begin{array}{l} {D_{i}}^{t+1}\in {\mathbb{R}}^{B,1,H,W}=\lambda_{i0}{D_{i}}^{0}+\underset{j\in N\left(k\right)}{\sum }\lambda_{ij}{D_{i}}^{t}, \end{array}$$

where *t* is the number of iterations, λ is the weight, and *N*(*k*) is the range of the neighborhood.

PE-Net was proposed to replace the pixel operation with the tensor operation. The formula was equivalent to


(9) $$\begin{array}{l} {D_{i}}^{t+1}\in {\mathbb{R}}^{B,1,H,W}=\chi \left({A_{i}}^{0},0\right)\chi \left({D_{i}}^{0},0\right)+\underset{j\in N\left(k\right)}{\sum }\chi \left(A_{i},j\right)\chi \left({D_{i}}^{t},j\right), \end{array}$$

where χ(·) is the vector translation operation and *A* is the affinity.

Further, we set *p* different convolution kernels for parallel space propagation and summed the results of each branch. The result of multiple serial and continuous convolution kernels at each scale ${D_{ip}}^{t+1}$ can be expressed as


(10) $$\begin{array}{l} {D_{ip}}^{t+1}\in {\mathbb{R}}^{B,1,H,W}=\chi \left({A_{ip}}^{0},0\right)\chi \left({D_{ip}}^{0},0\right)+\\ \underset{j_{p}\in N_{p}\left(k\right)}{\sum }\chi \left(A_{ip},j_{p}\right)\chi \left({D_{ip}}^{t},j_{p}\right) \end{array}$$

**Figure 4:**
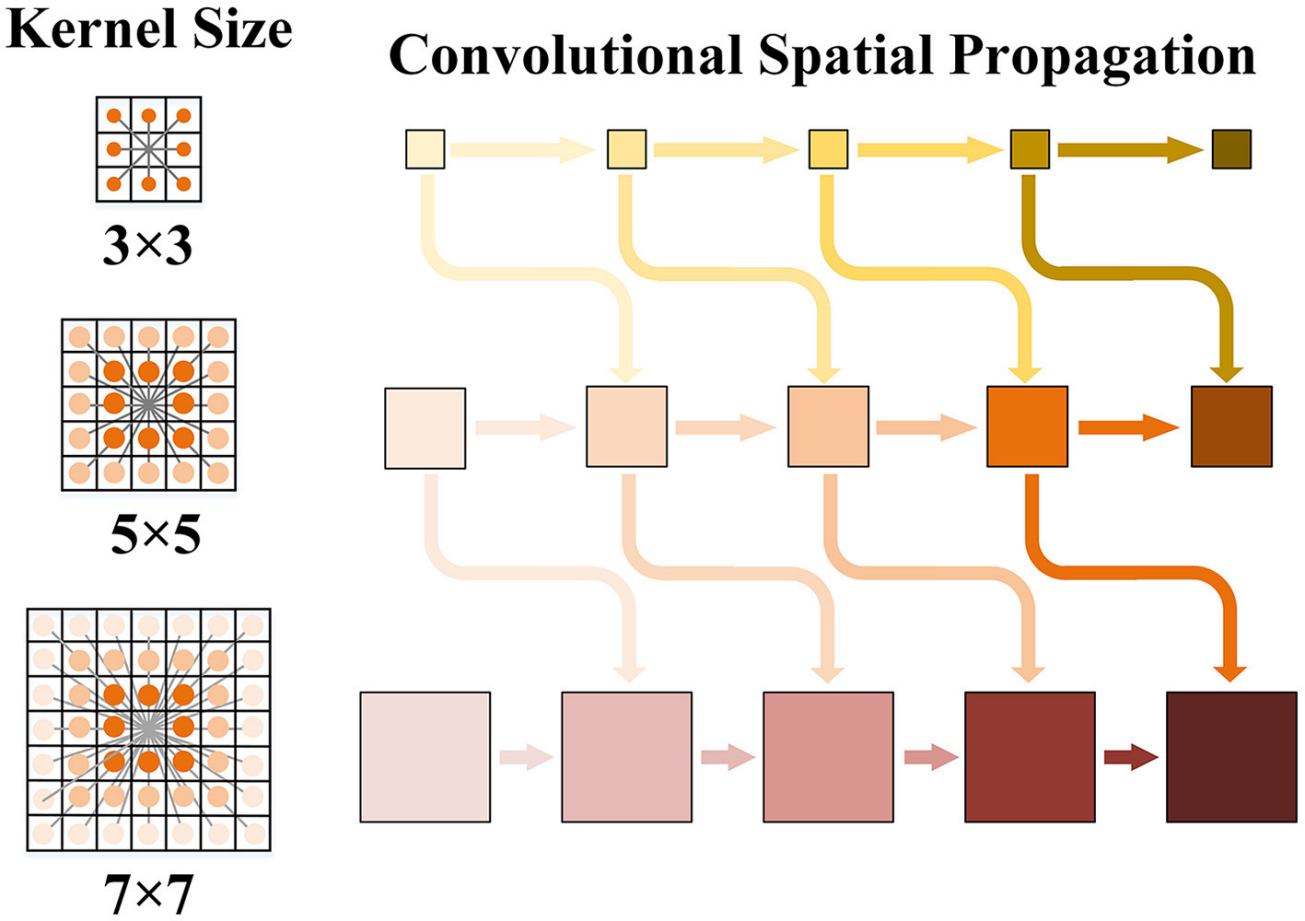
Schematic diagram of kernel convolution space propagation. Three convolution kernels are taken as examples. The local depth information is enhanced with the horizontal arrows propagating to the right respectively. There is cross-scale connection guidance between cores of different sizes.

However, different convolution kernels were related to each other, as they convolved around the same range of intersections. Choosing small convolution kernels may enhance local details. However, selecting too large a convolution kernel may lead to more unstable details and blurred edges. Therefore, we fused convolution kernels of different sizes into different branch kernels using the interleaved mode. We built the kernel-connected convolutional space propagation optimization module, which is shown in Figure 4. The spatial propagation networks of different convolution kernels were no longer parallel algorithms but were cross-connected with each other. We integrated small convolution kernels into large convolution kernels to guide the effect of convolution.

The branch consists of the convolution of itself and other branches. The improved convolution results at each scale ${{\hat{D}}_{ip}}^{t+1}$ can be expressed as


(11) $$\begin{array}{l} {{\hat{D}}_{ip}}^{t+1}\in {\mathbb{R}}^{B,1,H,W}=\chi \left({A_{ip_{q}}}^{0},0\right)\chi \left({D_{ip_{q}}}^{0},0\right)+\\ \underset{j_{p}\in N_{p}\left(k\right)}{\sum }\chi \left(A_{ip}+A_{ip_{q}},j_{p_{q}}\right)\chi \left({D_{ip_{q}}}^{t},j_{p_{q}}\right), \end{array}$$

where *q* is the convolution level of the other branches. When *q* is 0, it is the same as the original formula. The final dense-depth image $D_{fin}\in {\mathbb{R}}^{B,1,H,W}$ is obtained after refinement.

## 4. Experimental results and discussion

### 4.1. Dataset and setup

KITTI: We set up the experiment on the KITTI to verify the effectiveness of deep information processing for autonomous driving (Geiger et al., 2013). The data were collected using a Velodyne HDL-64E rotating 3D laser scanner and two PointGray Flea2 color cameras. It provided color images and corresponding sparse-depth images. Sparse-depth images were obtained by projecting 3D LiDAR points onto the corresponding image frames. In addition, a sparse-depth image had approximately 5% of valid pixels. A ground-truth, dense-depth image had approximately 16% of valid pixels. KITTI contained 43 k image pairs for training, 3 k for verification, and 1 k for testing. We split the validation set into 1 k pairs for validation and 1 k pairs for testing. Because of the uniqueness of autonomous driving, there was no depth information in the upper part of the image. Therefore, the 1,216 × 352 full-resolution images in the dataset were cropped from the bottom to 1,216 × 256.

p-KITTI: We proposed pre-processing binocular data from KITTI to simulate the missing effect in the real scene. Random *s* × *s* pixels of the binocular images were covered with a black mask. The other parameters of the images in the dataset remained unchanged. For example, if *s* = 100, it meant that 1.6% of the effective pixels were invalid.

Setup: We trained our network using Pytorch on one NVIDIA 2080 Ti GPU and chose the common settings without any improvements. We set the loss function as L1 loss, the optimizer as RMSProp, the constant learning rate as 1 × 10^−3^, and the batch size as 8. In addition, the image size for training was 512 × 256 images with random cropping.

Metric: MAE stands for the mean absolute error. It is the mean of the distance between the model's predicted value and the true value. It has a faster convergence, a more stable gradient, and a relatively robust solution. MSE stands for the mean square error. It refers to the mean squared difference between the predicted value of the model and the real sample value. Because its penalty is squared, it is sensitive to outliers. IMAE stands for the inverse mean absolute error. IRMSE stands for the inverse root mean square error. MARE stands for the mean absolute relative error. FPS refers to the number of frames per second. Error_1px, Error_2px, and Error_3px are errors of 1, 2, and 3 pixels, respectively. They can be expressed as


(12) $$\begin{array}{l} MAE=\frac{1}{n}\overset{n}{\underset{p\in P_{v}}{\sum }}|D_{p}^{gt}-D_{p}|, \end{array}$$

(13) $$\begin{array}{l} MSE=\frac{1}{n}{\overset{n}{\underset{p\in P_{v}}{\sum }}\left(D_{p}^{gt}-D_{p}\right)}^{2}, \end{array}$$

(14) $$\begin{array}{l} RMSE=\sqrt{\frac{1}{n}{\overset{n}{\underset{p\in P_{v}}{\sum }}\left(D_{p}^{gt}-D_{p}\right)}^{2}}, \end{array}$$

(15) $$\begin{array}{l} IMAE=\frac{1}{n}\overset{n}{\underset{p\in P_{v}}{\sum }}|\frac{1}{D_{p}^{gt}}-\frac{1}{D_{p}}|, \end{array}$$

(16) $$\begin{array}{l} IRMSE=\sqrt{\frac{1}{n}{\overset{n}{\underset{p\in P_{v}}{\sum }}\left(\frac{1}{D_{p}^{gt}}-\frac{1}{D_{p}}\right)}^{2}}, \end{array}$$

(17) $$\begin{array}{l} MARE=\frac{1}{n}\overset{n}{\underset{p\in P_{v}}{\sum }}\frac{\left|D_{p}^{gt}-D_{p}\right|}{D_{p}^{gt}}, \end{array}$$

and


(18) $$\begin{array}{l} FPS=\frac{1}{Time}, \end{array}$$

where *P*_*v*_ is the valid pixels, $D_{p}^{gt}$ is the true value of the pixel *p*, *D*_*p*_ is the predicted value of the pixel *p*, and *n* is the number of points.*Time* is the running time of inference.

### 4.2. Results of the experiment

#### 4.2.1. KITTI dataset

**Figure 5:**
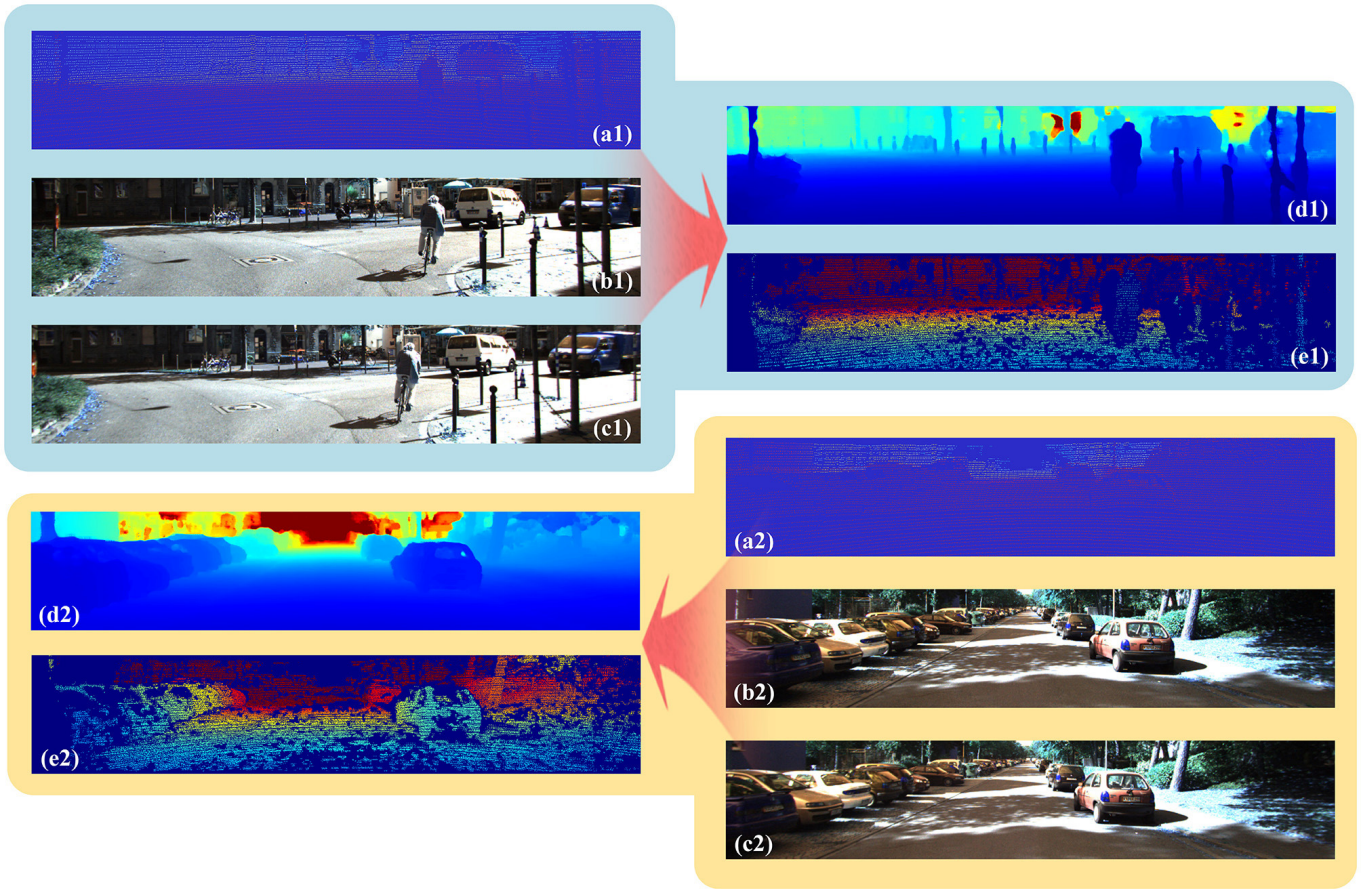
Results of our network on KITTI. **(a)** is the sparse point cloud from the LiDAR, **(b)** is the left image of the binocular camera, **(c)** is the right image of the binocular camera, **(d)** is the dense depth image completed by our network, and **(e)** is the ground truth of the dense point cloud.

The effect of deep completion based on LiDAR-stereo for autonomous driving is shown in Figure 5. The depth image is more dense and accurate due to information from multiple sources. In the first image of the road ahead, cyclists, vans, and pillars were evident on both sides of the road. In the second image, there were many cars parked to the left and right, which was clear in the depth image. The results of the comparison with other networks are shown in Tables 1, 2. The difference between the methods in Tables 1, 2 is running time. The speeds of the methods in the two tables were set at different levels.

**Table 1:** Comparison of real-time network effect on KITTI.

**Model**	**Input**	**IRMSE**	**IMAE**	**RMSE**	**MAE**	**Rank**	**Time (ms)**	**FPS (Hz)**
PASM-Net	Stereo	3.75	1.69	2.3082	0.6991	7.000	0.061	16.41
S2D	LiDAR	3.21	1.35	0.9544	0.2585	3.375	0.040	25
HMS-Net		2.93	1.14	0.9375	0.2585	4.625	**0.020**	**50**
S2D(RGB)	LiDAR+Mono	2.80	1.21	**0.8147**	0.2499	2.250	0.080	12.50
SLS-Fusion		2.72	1.28	0.8452	0.3074	3.250	-	-
HDE-Net	LiDAR+Stereo	3.39	1.38	2.0212	0.5005	6.000	0.045	22.22
RLS-Net(Ours)		**1.78**	**0.82**	0.9036	**0.2289**	**1.500**	0.067	14.93

The bold numbers are the best.

**Table 2:** Comparison of fusion network results on KITTI.

**Model**	**Input**	**IRMSE**	**IMAE**	**RMSE**	**MAE**	**Rank1**	**Rank2**	**Time(s)**	**FPS (hz)**
PSM-Net	Stereo	4.27	3.02	2.9328	1.2398	-	-	0.358	2.79
GA-Net		3.08	1.31	1.5292	0.4487	-	-	2.439	0.41
AA-Net+		2.97	1.23	1.3966	0.3934	-	-	0.234	4.27
CSPN	LiDAR + Mono	2.93	1.15	1.0196	0.2795	6.000	-	1.000	1
CSPN++		2.07	0.90	0.7437	0.2093	2.875	-	0.200	5
ACM-Net		2.08	0.90	0.7449	0.2061	3.125	-	0.200	5
NLSPN		1.99	0.84	0.7417	0.1996	**1.500**	-	0.220	4.55
Guide-Net		2.36	0.99	0.8578	0.2340	4.750	-	0.153	6.51
LiStereo	LiDAR + Stereo	2.19	1.10	0.8322	0.2839	-	5.750	-	-
VPN		1.88	0.99	0.6362	0.2051	-	3.000	1.408	0.71
SLF-Net		1.77	0.88	**0.6411**	**0.1970**	**-**	**2.250**	0.163	6.14
CCVNorm		**1.40**	**0.81**	0.7493	0.2525	-	2.500	1.011	0.99
SDC-Net		2.04	0.82	0.7524	0.2384	-	3.875	0.34	2.94
RLS-Net(Ours)		1.78	0.82	0.9036	0.2289	2.750	3.625	**0.068**	**14.71**

The bold numbers are the best.

The comparison of accuracy on KITTI between our network and other real-time networks is shown in Table 1. As can be observed, the depth completion improved with input diversification. Only LiDAR input had good real-time performance. The FPS could reach more than 25. However, the accuracy was very low. LiDAR-monocular input can serve as a balance with less promotion. Because of the additional information in a color image, the metrics of S2D(RGB) decreased by approximately 0.41 (13%), 0.21 (10%), 0.1397 (15%), and 0.0086 (3%), respectively. However, the speed was reduced by half. It was difficult to meet the real-time requirements of LiDAR-stereo input. Speed and accuracy cannot be ensured without effective fusion and optimization. Therefore, we comprehensively considered the factors affecting speed and the optimization methods to improve global and local accuracy. Our network achieved higher accuracy and still has real-time performance of 14.93 Hz. It has abilities that other LiDAR-stereo networks do not possess. Compared with S2D, the metrics of our network decrease by 1.43 (45%), 0.53 (39%), 0.0508 (5%), and 0.0296 (11%), with the 0.027 s going up. In addition, we used the Friedman test to compare the effects of these networks and validate the statistical significance of the above results (Shang et al., 2021; Yuan et al., 2022). We summarized each network's average rank in terms of the four metrics in Table 1. The results showed that our network achieves the best results using real-time methods.

Other LiDAR-mono and LiDAR-stereo networks focus more on improving accuracy. The comparison of accuracy is shown in Table 2. Our network had an absolute advantage in speed. Some metrics of our real-time network were better than those of some non-real-time networks. The metrics of CCVNorm had the best performance. For the closer metrics, IMAE decreased by approximately 0.01(1%), and MAE decreased by ~0.0236(9%) compared to our method. However, the running time of our network was 0.943s shorter than that of CCVNorm. Our network was 15 times faster. For the statistical analysis, Rank 1 represented a statistical comparison of our network with other LiDAR-Mono networks. Rank 2 represented a statistical comparison of our network with other LiDAR-stereo networks. The results of our network are in the middle of the non-real-time algorithm ranking. However, it is worth noting that our network ran much faster than other networks.

#### 4.2.2. P-KITTI dataset

In addition, another vital advantage of the fusion of binoculars and point clouds was the ability to avoid sensor defects and environmental impacts. The official KITTI dataset selects images and point clouds from complete and clear datasets. It was not affected by any unforeseen circumstances. The results with the original data cannot reflect its universality and robustness. Therefore, we pre-processed the images of KITTI and assumed that the defects of the sensor led to large holes in the binocular images. We demonstrated the advantages of multi-sensor fusion in Table 3. The results of LiDAR-Stereo fusion were better than those of a single sensor with the problem data. On some measures, the gap was even nearly 100 times greater. Multi-source sensors greatly improved the robustness of the algorithm.

**Table 3:** Comparison results on p-KITTI.

**Model**		**IRMSE**	**IMAE**	**RMSE**	**MAE**
Stereo	Image_2_cut	10.873	3.424	3.476	1.009
	Difference	−8.408	−2.137	−2.080	−0.556
	Image_3_cut	24.061	5.948	3.810	1.119
	Difference	−21.596	−4.661	−2.414	−0.6664
	Image_2+3_cut	13.751	4.528	18.289	4.945
	Difference	−11.286	−3.241	−16.893	−4.492
LiDAR+Stereo	Image_2_cut	1.794	0.834	0.970	0.250
	Difference	−0.014	−0.014	−0.066	−0.182
	Image_3_cut	1.807	0.838	0.942	0.251
	Difference	−0.027	−0.018	−0.038	−0.183
	Image_2+3_cut	1.952	0.861	1.659	0.269
	Difference	−0.172	−0.041	−0.755	−0.201

**Figure 6:**
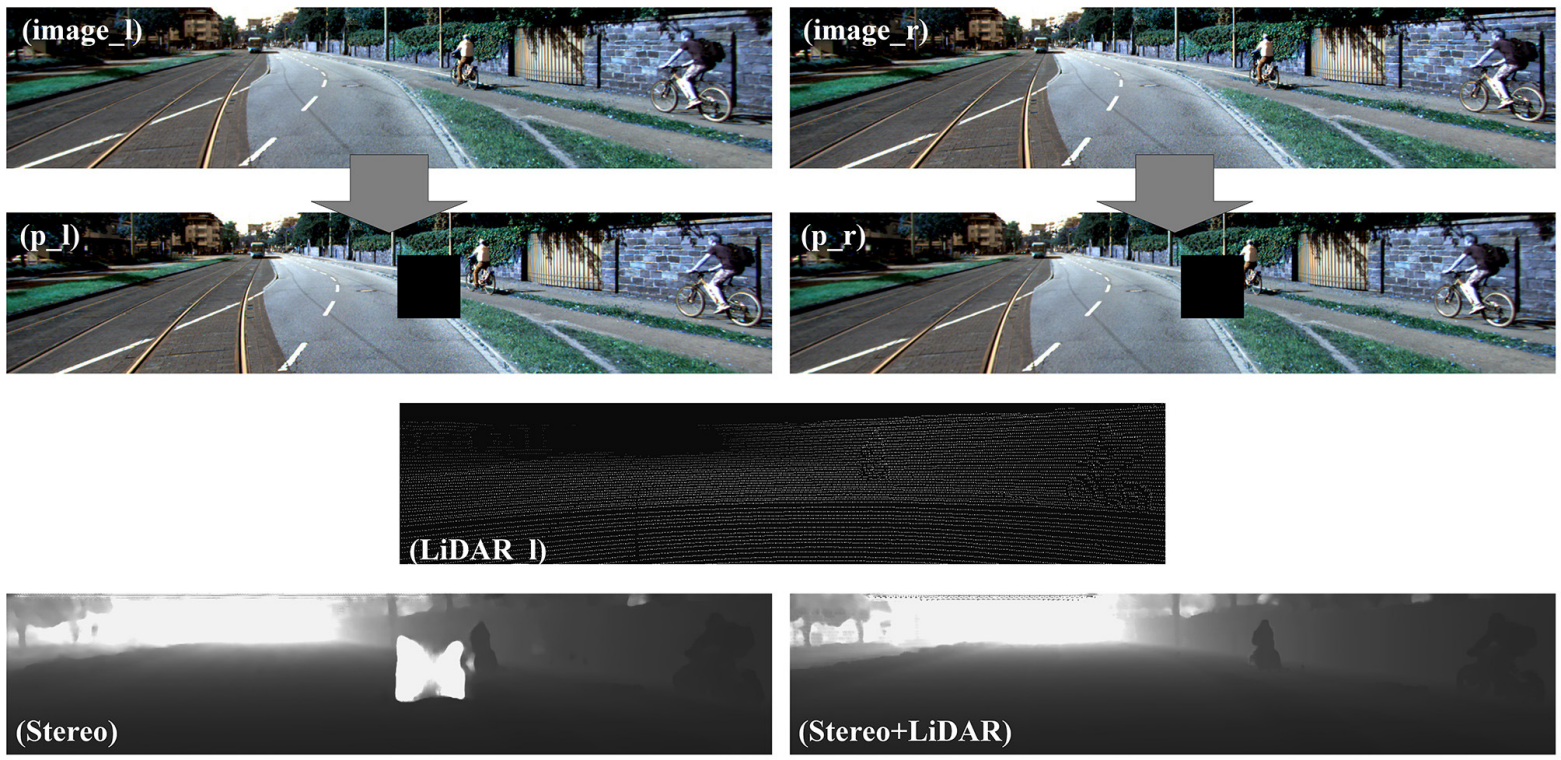
Results of our network using problem data. p_l and p_r were obtained after the left and right images were pre-processed. Stereo is the depth image obtained by stereo matching. Stereo+LiDAR is the depth image obtained by our network based on LiDAR-Stereo.

More details are shown in Figure 6. When the problem data were input into the stereo-matching network, the single sensor had a strong dependence on the data. The depth information cannot be compensated for by the network with pixel loss. As a result, the depth image appeared with large white holes and was affected by the perception of the environment. However, our networks based on multi-source sensors can greatly improve the defect. The depth map was still nicely completed because of the addition of information regarding LiDAR.

### 4.3. Results of the experiment

#### 4.3.1. Injection-guided initialization

There were various multi-source sensor fusion methods. We compared common input and feature fusion methods with our injection fusion. As shown in Table 4, the input and feature fuses produced worse results. It was caused by the lack of accurate correspondence between different sources of information. 3D convolution can alleviate it but at a huge time cost. Information mismatches are more common in real-life situations. Therefore, if we want to ensure real-time performance, these two fusion methods are not applicable. Injection fusion has no such problem. We gradually learned how to match multiple sources of information through the network rather than match information at the beginning. The time only increased by 0.002 s to integrate LiDAR information into binocular information. However, the metrics of the network decreased by 0.2347 (10%), 0.2194 (17%), 0.146 (10%), 0.0878 (19%), and 0.0037 (19%), respectively.

**Table 4:** Results of the injected guided initialization and updating module.

**Model**	**IRMSE**	**IMAE**	**RMSE**	**MAE**	**MARE**	**Time(s)**
RLS-Net_ori	2.4654	1.2870	1.3961	0.4526	0.0191	**0.062**
+ input fusion	3.9963	1.6608	1.6104	0.4420	0.0213	0.063
+feature fusion	4.3087	2.1358	1.8819	0.5987	0.0285	0.066
+ injected fusion	2.2307	1.0676	1.2501	0.3648	0.0154	0.064
+ updating	**1.9252**	**0.9301**	**0.9643**	**0.2880**	**0.0128**	0.066

The bold numbers are the best.

**Figure 7:**
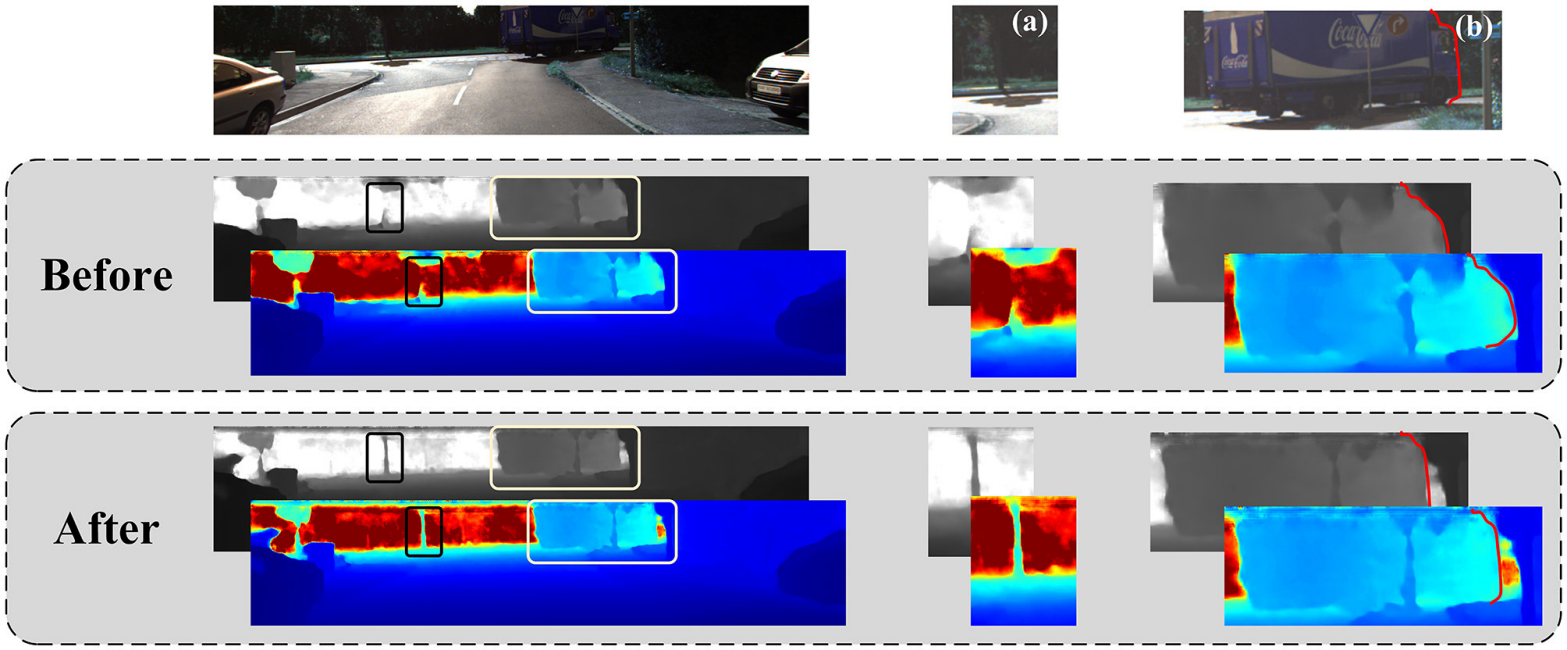
The effects of the injected guided initialization module. The first row is the colored left image. The second line is the result before adding the module. The third line is the result of adding the module. **(a)** and **(b)** are two details that are cropped and enlarged for obvious display.

As shown in Figure 7, the cross-sensor 3D information was fused effectively. The trunk of the color image was buried in (a) by a background of similar color. Therefore, the binocular images did not match the depth information of the trunk well. Because the car was far away and had no clear shape, it was integrated with the environment in (b) in the depth image. However, binocular images are no longer the only criterion for judgment with the additional point cloud information. We obtained a clear view of the tree trunk and the exact position of the car's head.

#### 4.3.2. Injection-guided updating

**Figure 8:**
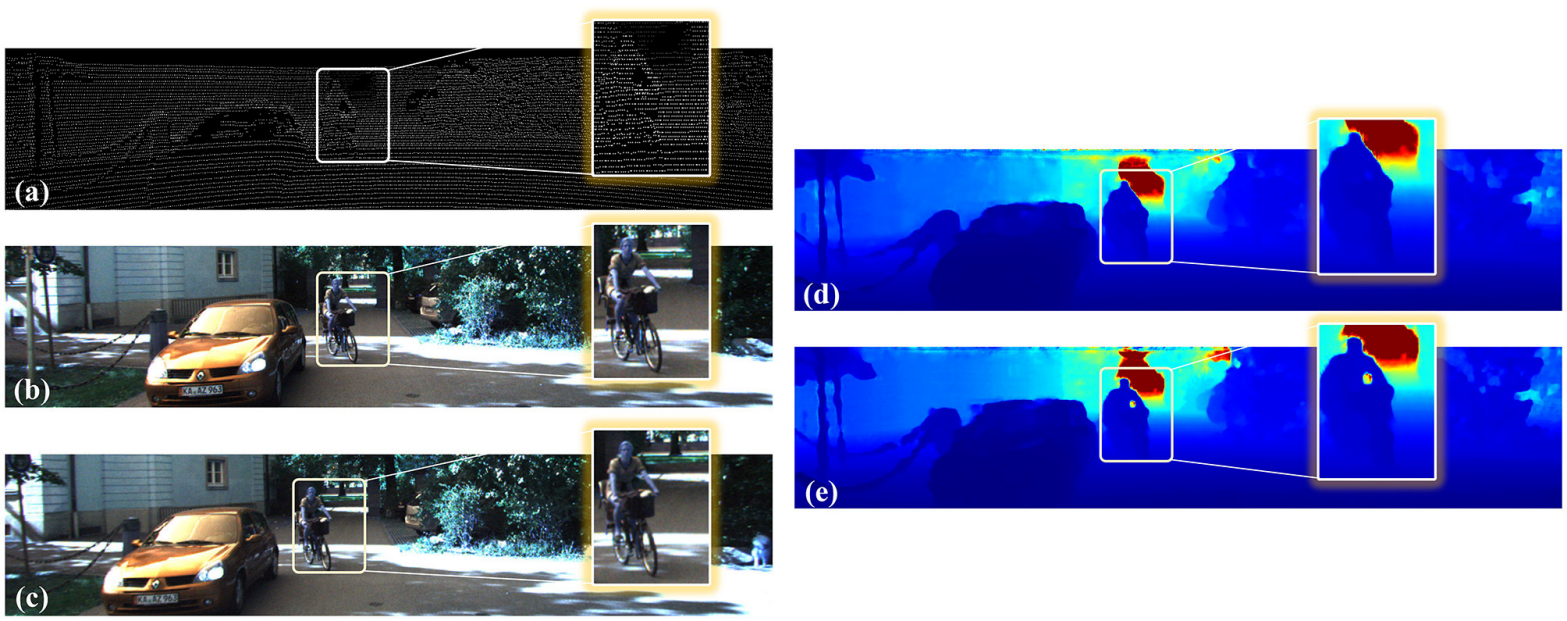
The effects of the injected guided updating module. **(a)** is the sparse point cloud. **(b, c)** are the left and right image pairs. **(d)** is the result before adding the module, and **(e)** is the result after adding the module.

The effect of our network after multiple injection-guided updates is shown in Table 4. The time of our network increased by 0.002 s, but the metrics of the network decreased by 0.3055(14%), 0.1375(13%), 0.0768(21%), and 0.0026(17%), respectively. The improvement was remarkable. In this step, the coarse-depth image was continuously updated with the guidance of point cloud injection. As shown in Figure 8, the connection between the handlebar and the hand was ignored because the image was affected by illumination and occlusion. However, it was evident in the sparse point clouds. After the injection guidance update, the depth was gradually optimized and updated with the fusion of the network. Details of the people and bikes were clearly shown.

In addition, as shown in Table 5, the pixel error was greatly reduced because of the point cloud injection. Finally, the pixel errors of the network decreased by 0.0030 (52%), 0.0050 (52%), and 0.0187 (57%), respectively.

**Table 5:** Results of the injected guided updating module.

**Model**	**Error_3px**	**Error_2px**	**Error_1px**
RLS-Net_ori	0.0058	0.0105	0.0327
+ injected fusion	0.0041	0.0071	0.0179
+ updating	**0.0028**	**0.0050**	**0.0140**

The bold numbers are the best.

#### 4.3.3. Kernel-connected depth refinement

A kernel-connected convolutional spatial propagation network requests iteration. The more iterations, the longer the time. Therefore, it was impossible to increase The Times blindly if the real-time performance was guaranteed. In addition, as shown in Table 6, the best results were achieved when the number was 16. We believe that the error information was magnified because of the excessive number of iterations, which affected the quality of the image. The time cost of our kernel connection optimization module increased by 0.001 s, but the metrics of the network decreased by 0.1455 (8%), 0.1139 (12%), 0.0607 (6%), 0.0591 (21%), and 0.002 (16%), respectively.

**Table 6:** Results of the kernel-connected depth refinement module.

**Model**	**IRMSE**	**IMAE**	**RMSE**	**MAE**	**MARE**	**Time(s)**
RLS-Net_updating	1.9252	0.9301	0.9643	0.2880	0.0128	**0.066**
+ KCSPN(12)	1.8910	0.8585	**0.8787**	0.2380	0.0114	0.067
+ KCSPN(16)	**1.7797**	**0.8162**	0.9036	**0.2289**	**0.0108**	0.067
+ KCSPN(20)	1.8259	0.8192	1.3711	0.2309	0.0109	0.068

The bold numbers are the best.

**Figure 9:**
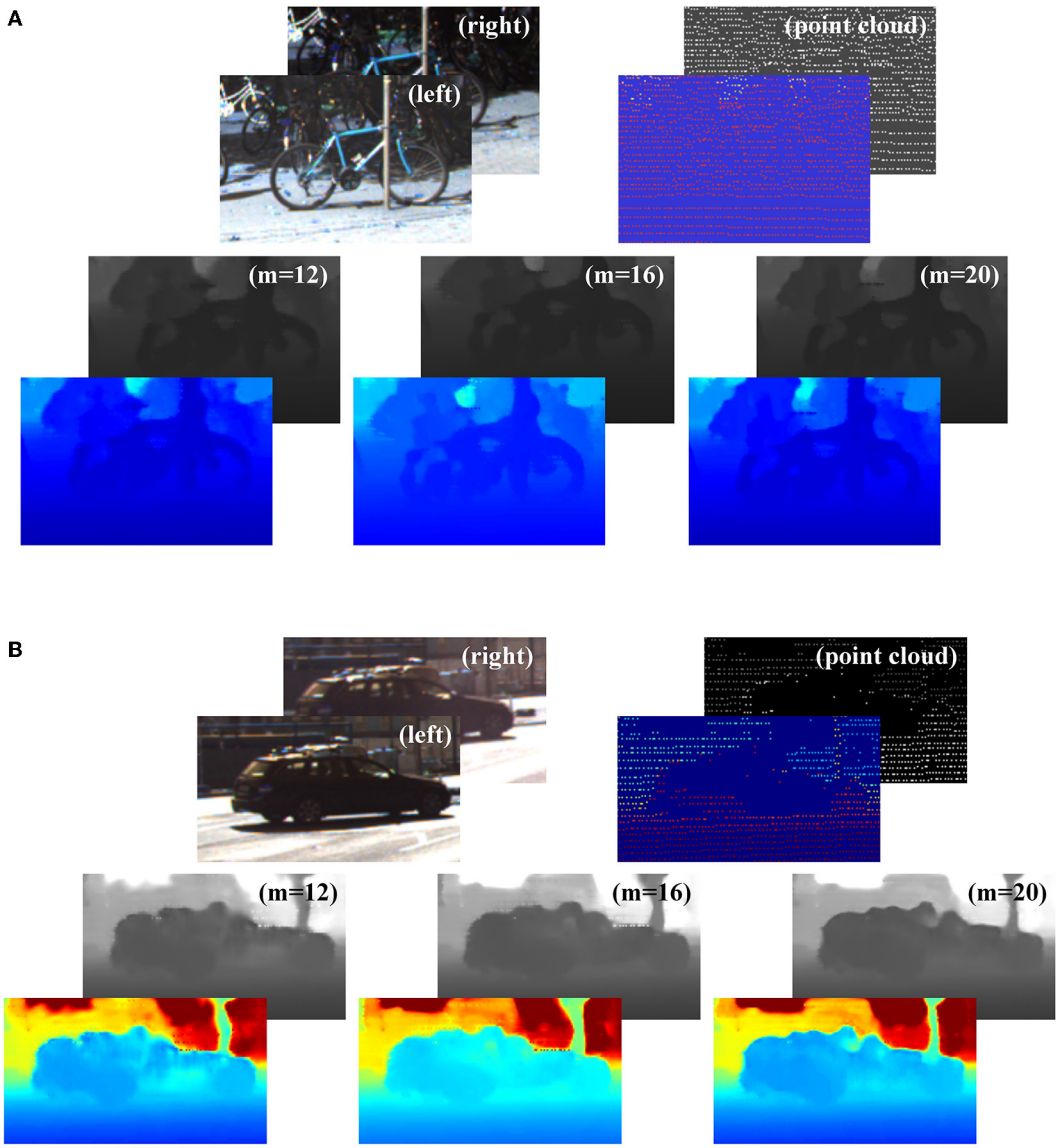
The effects of the injected guided updating kernel-connected depth refinement module. **(A)** is a bicycle and **(B)** is a car, both of which are specific targets for 3D information acquired by sensors. The top left image pair is binocular images. The top right is the point cloud. The following three images are the fine-depth images obtained at iterations 12, 16, and 20, respectively. The overlapped image at the bottom left is their false-color image.

As shown in Figure 9, (a) is a bicycle on the side of the road and (b) is a driving car. We could discover it intuitively from the color image. However, we could hardly recognize it from the background in the point cloud of the LiDAR. As shown in Figure 9, *m* was the number of iterations. When *m* = 16, the color image was better than when *m* = 12 and *m* = 20. As *m* increased, the proportion of point clouds gradually increased in the fusion process. The diversity of information was enhanced, and the target shape was more prominent. However, if the proportion of point clouds was too large, the accuracy of the fusion depth of the target was affected, and the result was excessively dependent on point clouds. Therefore, we chose *m* = 16 to optimize the depth image.

## 5. Conclusion

Aiming at the fusion problem of multi-source sensors for autonomous driving, we proposed a real-time LiDAR-Stereo depth information completion network. We initialized and updated injection-guided tile hypotheses for multiple network phases instead of 3D convolution. It was not only more conducive to the global fusion of multi-source information but also greatly reduced the cost of time and space. In addition, the spatial propagation network based on kernel connections effectively refined the local depth. A series of ablation experiments demonstrated the effectiveness of our module. Our network was proven to be effective and high-speed on the KITTI and p-KITTI datasets.

## Data availability statement

The original contributions presented in the study are included in the article/Supplementary material, further inquiries can be directed to the corresponding author.

## Author contributions

MW and YZ: core, architecture, conceptualization, and writing. MZ and JW: funding acquisition. JS: revising and validation. All authors contributed to the article and approved its submitted version.

## Appendix

**Table:** Table of Notation.

**Symbol**	**Explanation**
**A**	affinity
**b**	baseline
**B**	Batch size
**C**	the number of channels
***conv*()**	convolution
${d_{i}}^{0}$	the initial binocular disparity
${d_{ix}}^{0}$	the ladder difference between the two in the *x* direction
${d_{iy}}^{0}$	the ladder difference between the two in the *y* direction
***D*_*fin*_**	the final depth image
$D_{p}^{gt}$	the true value of the pixel *p*
***D*_*p*_**	the predicted value of the pixel *p*
***D*_*ori*_**	the coarse-depth image
***D*_*i*_**	convolutional space propagation
**F**	focal length
***F*_*j*_**	the feature image with *j* resolution
***flow*()**	the wrap method of optical flow
**H**	height
**i**	scale
**L**	the left image
**m**	the times
**n**	the number of points
**N**	the number of resolution layers
***N*(*k*)**	the range of the neighborhood
**p**	the number of convolution kernels
***P*_*v*_**	the set of valid pixels
***pad*()**	fill
**q**	the convolution kernels
**R**	the right image
**ℝ**	the data structure of the feature
${s_{i}}^{0}$	the sparsity of the point cloud
***s* × *s***	the pixels of the left and right images
**t**	the number of iterations
${T_{i}}^{0}$	the initial tile hypothesis of scale*i*
${{Ṫ}_{i}}^{m}$	the result of self-updating
${\ddot{T}}_{i}^{m}$	the result of depth updating
${\overset{\mathrm{...}}{T}}_{i}^{m}$	the result of point cloud updating
**Time**	the running time of inference
**u**	the offset of the mapping
**W**	width
***win*()**	the local setting window
**Z**	the depth of the scene
${z_{i}}^{0}$	the initial depth of the binocular
$\sigma_{i}^{l}$	left feature image
$\sigma_{i}^{r}$	right feature image
$\sigma_{i}^{s}$	feature of the point cloud
**λ**	weight
**χ(·)**	the vector translation operation
