# Supplementary material for: Real-time depth completion based on LiDAR-stereo for autonomous driving
Source: Front Neurorobot. 2023 Apr 18;17:1124676. doi: 10.3389/fnbot.2023.1124676 (PMC10151502; doi:10.3389/fnbot.2023.1124676)
Supplement: Supplementary file 1 [file Data_Sheet_1.PDF]

Supplementary Material

Real-time Depth Completion Based on LiDAR-Stereo for Autonomous Driving

Ming Wei, Ming Zhu*, Yaoyuan Zhang, Jiarong Wang, Jiaqi Sun

*** Correspondence:** Corresponding Author: zhuming@ciomp.ac.cn

1 Supplementary Figures and Tables

1.1 Supplementary Figures

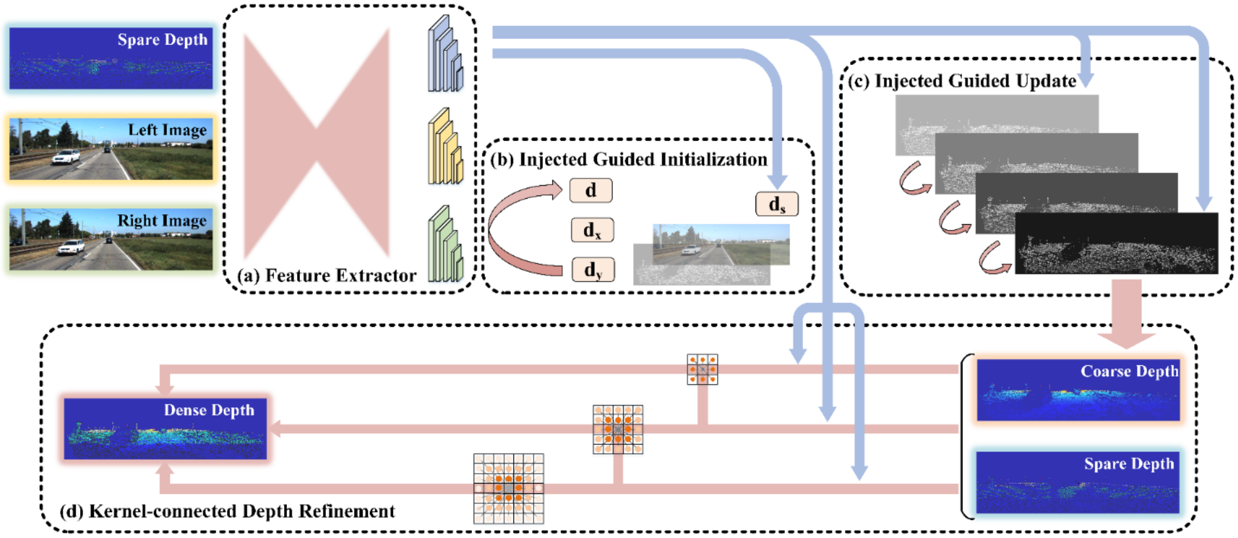

Supplementary Figure 1. The structure of our network. The inputs of the network are the sparse depth image from LiDAR and the image pair from the binocular camera. The output is the dense depth image. Initially, The multi-source images get the corresponding features through the feature extractor with shared weight. Then, the coarse depth is obtained by initializing and iteratively

updating the depth information under the guidance of the features of the point cloud. Finally, the depth is optimized in the refinement module to obtain the dense depth image.

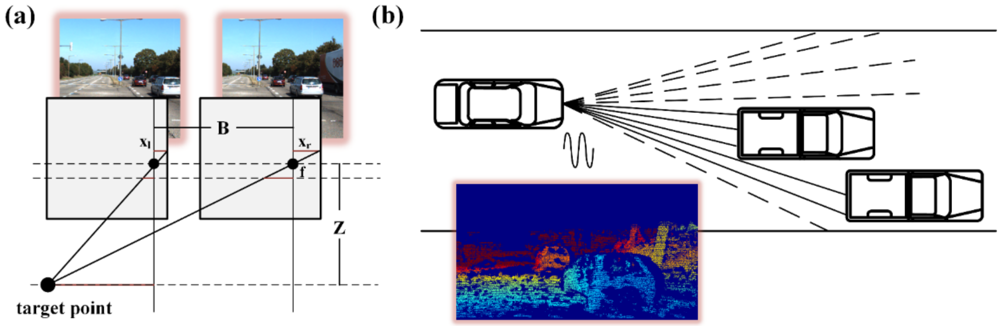

Supplementary Figure 2. Depth measurement model of stereo matching and point cloud scanning. (a) is the model of a binocular camera, the lower dot is the target point, and the upper two dots are the two cameras. (b) is the model of LiDAR, the lines are waves.

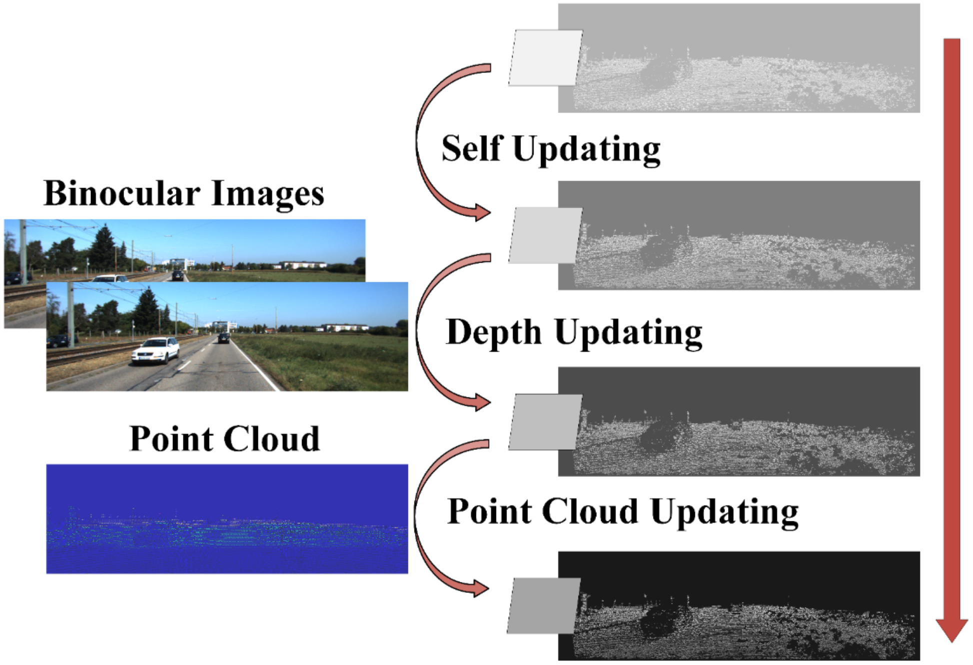

Supplementary Figure 3. Schematic diagram of triple updating. Tile hypotheses are the majority of feature representation and update for depth images. The arrow from top to bottom in the direction of

the update. The binocular image is injected in the depth updating. The point cloud is incorporated into the point cloud updating.

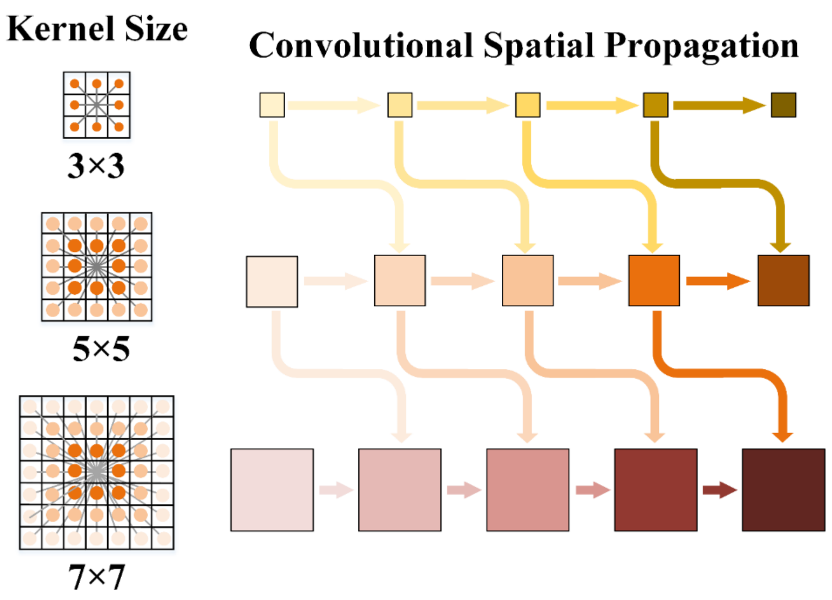

Supplementary Figure 4. Schematic diagram of kernel convolution space propagation. Three convolution kernels are taken as examples. The local depth information is enhanced with the

horizontal arrows propagating to the right respectively. There is cross-scale connection guidance between cores of different sizes.

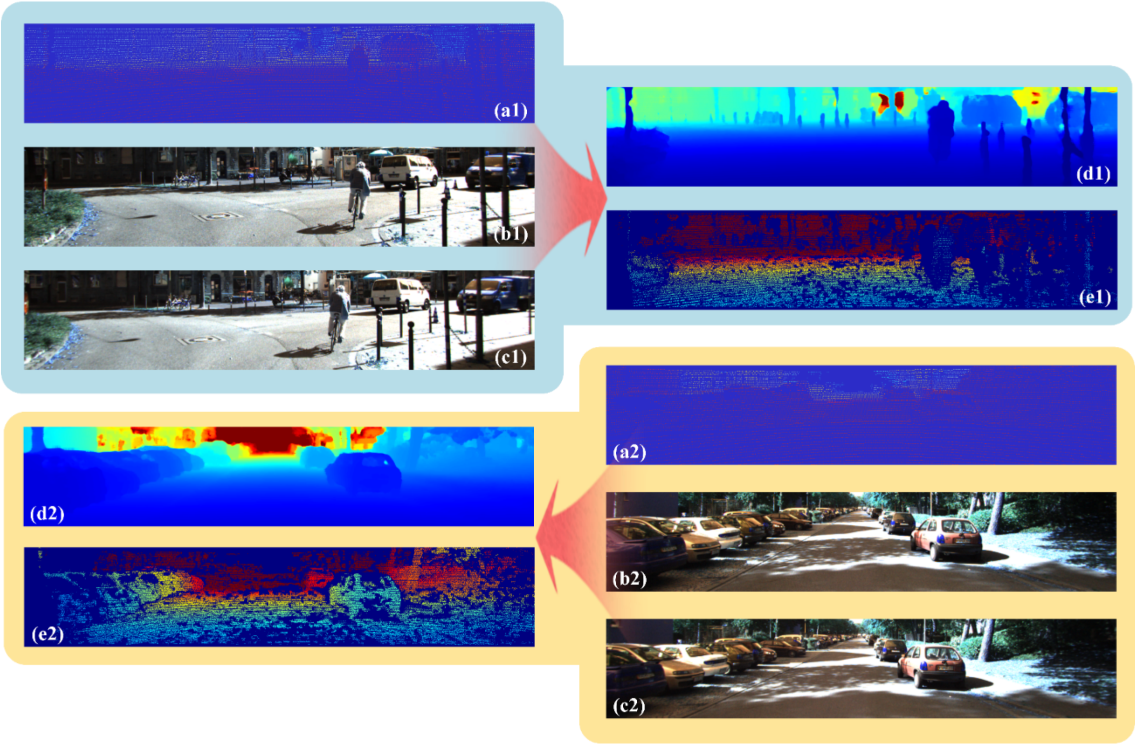

Supplementary Figure 5. Results of our network on KITTI. (a) is the sparse point cloud from the LiDAR, (b) is the left image of the binocular camera, (c) is the right image of the binocular camera,

(d) is the dense depth image completed by our network, and (e) is the ground truth of the dense point cloud.

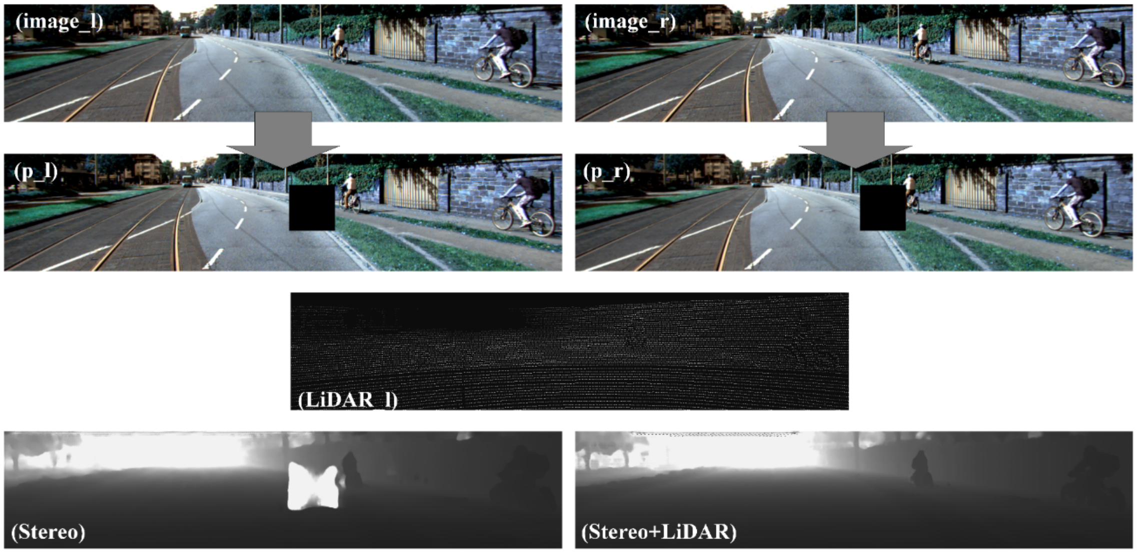

Supplementary Figure 6. Results of our network using problem data. p_l and p_r were obtained after the left and right images were pre-processed. Stereo is the depth image obtained by stereo matching. Stereo+LiDAR is the depth image obtained by our network based on LiDAR-Stereo.

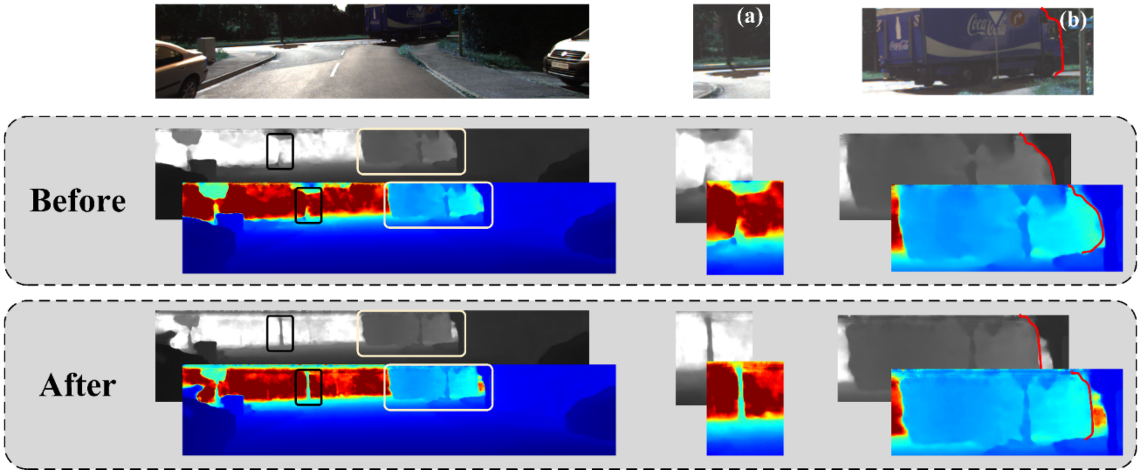

Supplementary Figure 7. The effects of the injected guided initialization module. The first row is the colored left image. The second line is the result before adding the module. The third line is the

result of adding the module. (a) and (b) are two details that are cropped and enlarged for obvious display.

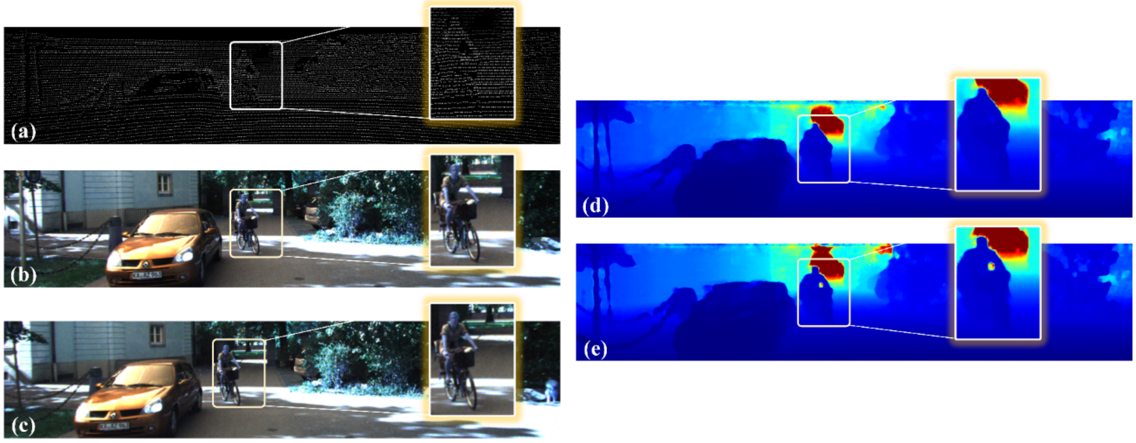

Supplementary Figure 8. The effects of the injected guided updating module. (a) is the sparse point cloud. (b) and (c) are the left and right image pairs. (d) is the result before adding the module, and (e) is the result after adding the module.

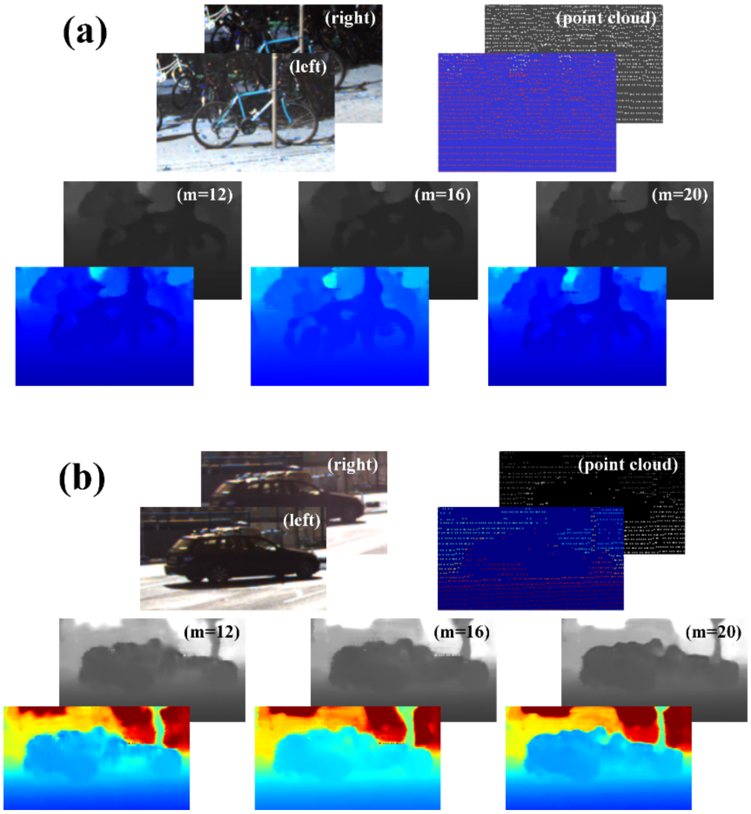

Supplementary Figure 9. The effects of the injected guided updating kernel-connected depth refinement module. (a) is a bicycle and (b) is a car, both of which are specific targets for 3D information acquired by sensors. The top left image pair is binocular images. The top right is the point cloud. The following three images are the fine-depth images obtained at iterations 12,16 and 20, respectively. The overlapped image at the bottom left is their false-color image.
